# *catena*-Poly[[bis­(1-allyl­imidazole)zinc(II)]-μ-phthalato-κ^2^ *O* ^1^:*O* ^2^]

**DOI:** 10.1107/S1600536810003892

**Published:** 2010-02-06

**Authors:** Rong-Xun Li, Qi-Ye Wu, Fa-Qian Liu

**Affiliations:** a School of Materials Science and Engineering, University of Science and Technology Beijing, Beijing 100083, People’s Republic of China

## Abstract

The structure of the title compound, [Zn(C_8_H_4_O_4_)(C_6_H_8_N_2_)_2_]_*n*_, exhibits polymeric zigzag chains extended along the *c* axis. The Zn^II^ ion is coordinated by two N [Zn—N = 2.008 (6) and 2.012 (6) Å] and two O [Zn—O = 1.959 (5) and 1.985 (5) Å] atoms in a distorted tetra­hedral geometry. Weak C—H⋯O inter­actions contribute to the crystal packing stability.

## Related literature

In the corresponding zinc compounds, [Zn(phthalato)(1-*H*-vinyl­imidazole)_2_] (Li *et al.*, 2007*a* ▶) and [Zn(phthalato)(1-*H*-ethyl­imidazole)_2_] (Li *et al.*, 2007*b* ▶), the Zn^II^ ions also have a distorted tetra­hedral environment.
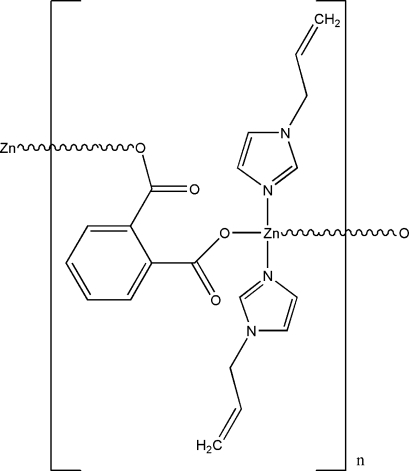


## Experimental

### 

#### Crystal data

[Zn(C_8_H_4_O_4_)(C_6_H_8_N_2_)_2_]
*M* *_r_* = 445.77
Orthorhombic, 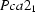

*a* = 10.675 (2) Å
*b* = 13.858 (3) Å
*c* = 13.610 (3) Å
*V* = 2013.4 (7) Å^3^
*Z* = 4
Mo *K*α radiation
μ = 1.25 mm^−1^
*T* = 293 K
0.20 × 0.10 × 0.10 mm

#### Data collection

Bruker SMART 1K CCD area-detector diffractometer
Absorption correction: multi-scan (*SADABS*; Sheldrick, 2004 ▶) *T*_min_ = 0.788, *T*_max_ = 0.885
3662 measured reflections
3078 independent reflections
2672 reflections with *I* > 2σ(*I*)
*R*_int_ = 0.013

#### Refinement

*R*[*F* ^2^ > 2σ(*F* ^2^)] = 0.063
*wR*(*F* ^2^) = 0.170
*S* = 1.01
3078 reflections
262 parameters
21 restraints
H-atom parameters constrained
Δρ_max_ = 0.86 e Å^−3^
Δρ_min_ = −0.94 e Å^−3^
Absolute structure: Flack (1983 ▶), 1148 Friedel pairs
Flack parameter: 0.01 (3)

### 

Data collection: *SMART* (Bruker, 2001 ▶); cell refinement: *SAINT* (Bruker, 2001 ▶); data reduction: *SAINT*; program(s) used to solve structure: *SHELXTL* (Sheldrick, 2008 ▶); program(s) used to refine structure: *SHELXTL*; molecular graphics: *SHELXTL*; software used to prepare material for publication: *SHELXTL* and local programs.

## Supplementary Material

Crystal structure: contains datablocks global, I. DOI: 10.1107/S1600536810003892/hg2640sup1.cif

Structure factors: contains datablocks I. DOI: 10.1107/S1600536810003892/hg2640Isup2.hkl

Additional supplementary materials:  crystallographic information; 3D view; checkCIF report

## Figures and Tables

**Table 1:** Hydrogen-bond geometry (Å, °)

*D*—H⋯*A*	*D*—H	H⋯*A*	*D*⋯*A*	*D*—H⋯*A*
C3—H3*A*⋯O2^i^	0.97	2.56	3.424 (10)	148
C5—H5*A*⋯O3	0.93	2.43	3.070 (10)	126
C6—H6*A*⋯O1	0.93	2.38	3.080 (11)	132
C9—H9*A*⋯O3^ii^	0.97	2.48	3.317 (11)	144

Symmetry codes: (i) 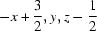; (ii) 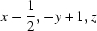.

**Fig. 1.**
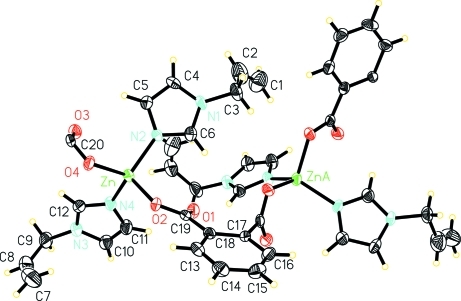
A portion of the polymeric chain in the title compound showing atomic numbering and 30% probability displacement ellipsoids [symmetry codes: (A) -x+3/2,y,z-1/2.

**Fig. 2.**
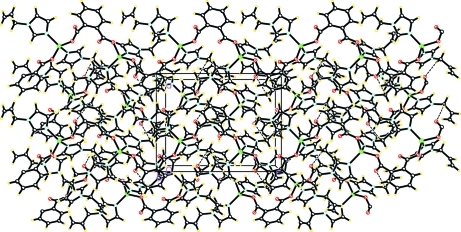
The packing of (I), viewed down the b axis.

## supplementary crystallographic information

### Comment

In the title compound,(I)(Fig. 1), The zinc(II) centers are bridged by the carboxylate group of *o*-phthalate and saturated by 1-allylimidazole. Each Zn^II^ ion is coordinated by two N [Zn—N2 = 2.012 (6),Zn—N4 = 2.008 (6) Å] and two O [Zn—O2 = 1.959 (5), Zn—O4 = 1.985 (5) Å] atoms in a distorted tetrahedral geometry. All these values agree well with those observed in [Zn(phthalato)(1-*H*-vinylimidazole)_2_] (Li *et al.*, 2007a) and [Zn(phthalato)(1-*H*-ethylimidazole)_2_] (Li *et al.*, 2007b). Each *o*-phthalate dianion acts as a bidentate ligand to bridge two Zn^II^ atoms through two monodentate carboxylate groups, building a zigzag infinate chain structure along the c axis. The metal-metal distances across each polymer backbone are 6.908 (6) Å.In the crystal, Weak C—H···O interactions contribute to the crystal packing stability.

In the corresponding zinc compounds, [Zn(phthalato)(1-*H*-vinylimidazole)_2_] (Li, *et al.*, 2007a) and [Zn(phthalato)(1-*H*-ethylimidazole)_2_] (Li, *et al.*, 2007b) the Zn^II^ ions have a distorted tetrahedral environment.

### Experimental

The reaction of ZnCl_2_(0.68 g, 5 mmol) with *o*-phthalic acid (0.83 g, 5 mmol) in an aqueous-methanol(3:1) solution(40 ml) at 363 K for 30 minutes produced a colorless solution, to which 1-allylimidazole (1.08 g,10 mmol) was added. The reaction solution was kept at room temperature after stirring for an hour at 363 K . Colorless crystals were obtained after a few days.

### Refinement

H atoms were positioned geometrically(C—H = 0.93-0.97 Å) and allowed to ride on their parent atoms with U_iso_(H) = 1.2 times U_eq_(C).

### Crystal data

**Table:**

[Zn(C_8_H_4_O_4_)(C_6_H_8_N_2_)_2_]	*F*(000) = 920
*M**_r_* = 445.77	*D*_x_ = 1.471 Mg m^−^^3^
Orthorhombic, *P**c**a*2_1_	Mo *K*α radiation, λ = 0.71073 Å
Hall symbol: P 2c -2ac	Cell parameters from 3003 reflections
*a* = 10.675 (2) Å	θ = 1.5–25.3°
*b* = 13.858 (3) Å	µ = 1.25 mm^−^^1^
*c* = 13.610 (3) Å	*T* = 293 K
*V* = 2013.4 (7) Å^3^	Block, colorless
*Z* = 4	0.20 × 0.10 × 0.10 mm

### Data collection

**Table:**

Bruker SMART 1K CCD area-detector diffractometer	3078 independent reflections
Radiation source: fine-focus sealed tube	2672 reflections with *I* > 2σ(*I*)
graphite	*R*_int_ = 0.013
thin–slice ω scans	θ_max_ = 25.3°, θ_min_ = 1.5°
Absorption correction: multi-scan (*SADABS*; Sheldrick, 2004)	*h* = 0→12
*T*_min_ = 0.788, *T*_max_ = 0.885	*k* = 0→16
3662 measured reflections	*l* = −16→16

### Refinement

**Table:**

Refinement on *F*^2^	Secondary atom site location: difference Fourier map
Least-squares matrix: full	Hydrogen site location: inferred from neighbouring sites
*R*[*F*^2^ > 2σ(*F*^2^)] = 0.063	H-atom parameters constrained
*wR*(*F*^2^) = 0.170	*w* = 1/[σ^2^(*F*_o_^2^) + (0.1*P*)^2^ + 1.7*P*] where *P* = (*F*_o_^2^ + 2*F*_c_^2^)/3
*S* = 1.01	(Δ/σ)_max_ < 0.001
3078 reflections	Δρ_max_ = 0.86 e Å^−^^3^
262 parameters	Δρ_min_ = −0.93 e Å^−^^3^
21 restraints	Absolute structure: Flack (1983), 1148 Friedel pairs
Primary atom site location: structure-invariant direct methods	Flack parameter: 0.01 (3)

### Special details

**Table:**

Geometry. All esds (except the esd in the dihedral angle between two l.s. planes) are estimated using the full covariance matrix. The cell esds are taken into account individually in the estimation of esds in distances, angles and torsion angles; correlations between esds in cell parameters are only used when they are defined by crystal symmetry. An approximate (isotropic) treatment of cell esds is used for estimating esds involving l.s. planes.
Refinement. Refinement of *F*^2^ against ALL reflections. The weighted *R*-factor wR and goodness of fit *S* are based on *F*^2^, conventional *R*-factors *R* are based on *F*, with *F* set to zero for negative *F*^2^. The threshold expression of *F*^2^ > σ(*F*^2^) is used only for calculating *R*-factors(gt) etc. and is not relevant to the choice of reflections for refinement. *R*-factors based on *F*^2^ are statistically about twice as large as those based on *F*, and *R*- factors based on ALL data will be even larger.

### Fractional atomic coordinates and isotropic or equivalent isotropic displacement parameters (Å^2^)

**Table:**

	*x*	*y*	*z*	*U*_iso_*/*U*_eq_	
Zn	0.80554 (7)	0.24867 (6)	0.17695 (11)	0.0404 (2)	
O1	0.6427 (6)	0.2741 (4)	0.0031 (4)	0.0527 (14)	
O2	0.6618 (4)	0.1729 (4)	0.1314 (3)	0.0460 (12)	
O3	1.0107 (5)	0.2747 (4)	0.3026 (4)	0.0499 (13)	
O4	0.8498 (5)	0.1737 (4)	0.2963 (3)	0.0475 (12)	
N1	1.0272 (6)	0.1987 (5)	−0.0680 (4)	0.0521 (17)	
N2	0.9385 (6)	0.2337 (5)	0.0733 (4)	0.0463 (15)	
N3	0.7033 (6)	0.5006 (4)	0.3164 (5)	0.0510 (16)	
N4	0.7491 (7)	0.3801 (4)	0.2205 (4)	0.0472 (14)	
C1	1.1212 (13)	0.2987 (10)	−0.2907 (9)	0.117 (4)	
H1A	1.0419	0.3048	−0.3185	0.140*	
H1B	1.1873	0.3358	−0.3143	0.140*	
C2	1.1396 (16)	0.2381 (9)	−0.2185 (9)	0.108 (4)	
H2A	1.2202	0.2342	−0.1927	0.130*	
C3	1.0418 (10)	0.1747 (7)	−0.1742 (6)	0.067 (3)	
H3A	0.9627	0.1841	−0.2079	0.081*	
H3B	1.0659	0.1076	−0.1813	0.081*	
C4	1.1200 (8)	0.2010 (7)	0.0013 (6)	0.064 (2)	
H4A	1.2048	0.1897	−0.0093	0.077*	
C5	1.0653 (8)	0.2229 (8)	0.0875 (6)	0.064 (2)	
H5A	1.1067	0.2296	0.1473	0.077*	
C6	0.9205 (9)	0.2199 (6)	−0.0230 (6)	0.054 (2)	
H6A	0.8433	0.2246	−0.0542	0.064*	
C7	0.5349 (13)	0.4544 (10)	0.4792 (11)	0.115 (4)	
H7A	0.5376	0.4124	0.4260	0.138*	
H7B	0.4812	0.4419	0.5315	0.138*	
C8	0.6023 (12)	0.5264 (10)	0.4805 (9)	0.103 (4)	
H8A	0.5944	0.5649	0.5361	0.124*	
C9	0.6961 (9)	0.5618 (7)	0.4052 (7)	0.071 (3)	
H9A	0.6737	0.6269	0.3858	0.085*	
H9B	0.7783	0.5644	0.4355	0.085*	
C10	0.6367 (8)	0.5131 (6)	0.2322 (6)	0.057 (2)	
H10A	0.5824	0.5636	0.2177	0.069*	
C11	0.6646 (7)	0.4394 (5)	0.1753 (7)	0.0564 (19)	
H11A	0.6315	0.4292	0.1130	0.068*	
C12	0.7688 (8)	0.4184 (6)	0.3080 (6)	0.0504 (19)	
H12A	0.8203	0.3924	0.3563	0.060*	
C13	0.4530 (7)	0.0680 (6)	0.0655 (6)	0.0515 (19)	
H13A	0.4738	0.0641	0.1317	0.062*	
C14	0.3601 (8)	0.0089 (6)	0.0297 (6)	0.057 (2)	
H14A	0.3168	−0.0323	0.0715	0.068*	
C15	0.3319 (8)	0.0118 (6)	−0.0695 (7)	0.058 (2)	
H15A	0.2695	−0.0281	−0.0945	0.070*	
C16	0.3937 (8)	0.0716 (6)	−0.1302 (6)	0.053 (2)	
H16A	0.3759	0.0702	−0.1971	0.064*	
C17	0.4853 (6)	0.1368 (5)	−0.0948 (5)	0.0379 (16)	
C18	0.5166 (6)	0.1329 (5)	0.0063 (5)	0.0386 (15)	
C19	0.6122 (7)	0.1995 (6)	0.0472 (5)	0.0424 (17)	
C20	0.9526 (7)	0.2027 (6)	0.3316 (5)	0.0380 (16)	

### Atomic displacement parameters (Å^2^)

**Table:**

	*U*^11^	*U*^22^	*U*^33^	*U*^12^	*U*^13^	*U*^23^
Zn	0.0443 (4)	0.0475 (4)	0.0294 (3)	0.0011 (4)	0.0035 (5)	0.0003 (4)
O1	0.056 (3)	0.062 (3)	0.041 (3)	−0.013 (3)	−0.002 (3)	0.002 (3)
O2	0.044 (3)	0.065 (4)	0.029 (2)	0.003 (3)	−0.008 (2)	−0.002 (2)
O3	0.051 (3)	0.057 (3)	0.042 (3)	−0.010 (3)	0.004 (3)	0.005 (3)
O4	0.045 (3)	0.066 (3)	0.032 (3)	−0.007 (3)	−0.008 (2)	0.004 (2)
N1	0.054 (4)	0.077 (5)	0.026 (3)	−0.004 (4)	0.012 (3)	−0.005 (3)
N2	0.039 (3)	0.070 (4)	0.030 (3)	0.003 (3)	0.008 (3)	0.004 (3)
N3	0.061 (4)	0.049 (4)	0.044 (3)	−0.002 (3)	0.002 (3)	−0.005 (3)
N4	0.058 (4)	0.043 (3)	0.040 (3)	0.003 (3)	−0.001 (3)	−0.011 (3)
C1	0.112 (8)	0.128 (8)	0.109 (8)	−0.030 (7)	−0.019 (7)	0.010 (7)
C2	0.134 (8)	0.117 (8)	0.072 (7)	−0.028 (7)	0.021 (7)	−0.005 (6)
C3	0.083 (6)	0.081 (6)	0.038 (4)	−0.016 (5)	0.015 (4)	−0.009 (4)
C4	0.047 (5)	0.092 (6)	0.054 (5)	0.012 (5)	0.016 (4)	−0.014 (5)
C5	0.048 (5)	0.102 (7)	0.041 (5)	0.006 (5)	−0.004 (4)	−0.006 (5)
C6	0.059 (5)	0.064 (5)	0.038 (4)	0.003 (4)	−0.002 (4)	0.003 (4)
C7	0.122 (11)	0.121 (10)	0.101 (10)	0.021 (7)	0.036 (9)	−0.004 (9)
C8	0.101 (9)	0.142 (11)	0.067 (7)	−0.005 (8)	−0.002 (7)	−0.042 (8)
C9	0.082 (7)	0.068 (6)	0.062 (6)	0.007 (5)	−0.013 (5)	−0.030 (5)
C10	0.068 (6)	0.052 (5)	0.052 (5)	0.012 (4)	−0.006 (4)	0.005 (4)
C11	0.072 (5)	0.055 (4)	0.042 (4)	0.010 (4)	−0.007 (5)	0.002 (5)
C12	0.047 (5)	0.057 (5)	0.047 (4)	0.008 (4)	−0.004 (4)	−0.006 (4)
C13	0.053 (5)	0.062 (5)	0.040 (4)	0.001 (4)	0.012 (4)	−0.002 (4)
C14	0.057 (5)	0.052 (5)	0.062 (5)	−0.010 (4)	0.016 (4)	0.003 (4)
C15	0.048 (5)	0.039 (4)	0.087 (7)	−0.009 (4)	−0.009 (4)	−0.009 (4)
C16	0.045 (5)	0.060 (5)	0.054 (5)	−0.001 (4)	−0.012 (4)	−0.003 (4)
C17	0.031 (4)	0.048 (4)	0.035 (4)	0.007 (3)	−0.003 (3)	−0.009 (3)
C18	0.038 (4)	0.044 (4)	0.034 (3)	0.009 (3)	0.003 (3)	0.003 (3)
C19	0.032 (4)	0.061 (5)	0.034 (4)	0.011 (4)	0.002 (3)	−0.011 (4)
C20	0.045 (4)	0.047 (4)	0.022 (3)	0.004 (4)	0.003 (3)	−0.007 (3)

### Geometric parameters (Å, °)

**Table:**

Zn—O2	1.959 (5)	C5—H5A	0.9300
Zn—O4	1.985 (5)	C6—H6A	0.9300
Zn—N4	2.008 (6)	C7—C8	1.230 (17)
Zn—N2	2.012 (6)	C7—H7A	0.9300
O1—C19	1.239 (9)	C7—H7B	0.9300
O2—C19	1.316 (9)	C8—C9	1.515 (16)
O3—C20	1.240 (9)	C8—H8A	0.9300
O4—C20	1.263 (9)	C9—H9A	0.9700
N1—C6	1.327 (11)	C9—H9B	0.9700
N1—C4	1.368 (11)	C10—C11	1.315 (11)
N1—C3	1.491 (9)	C10—H10A	0.9300
N2—C6	1.337 (10)	C11—H11A	0.9300
N2—C5	1.376 (11)	C12—H12A	0.9300
N3—C12	1.342 (9)	C13—C14	1.374 (11)
N3—C10	1.359 (10)	C13—C18	1.385 (10)
N3—C9	1.479 (10)	C13—H13A	0.9300
N4—C12	1.320 (10)	C14—C15	1.384 (12)
N4—C11	1.367 (10)	C14—H14A	0.9300
C1—C2	1.308 (9)	C15—C16	1.344 (12)
C1—H1A	0.9300	C15—H15A	0.9300
C1—H1B	0.9300	C16—C17	1.416 (11)
C2—C3	1.492 (16)	C16—H16A	0.9300
C2—H2A	0.9300	C17—C18	1.417 (9)
C3—H3A	0.9700	C17—C20^i^	1.509 (10)
C3—H3B	0.9700	C18—C19	1.483 (10)
C4—C5	1.346 (11)	C20—C17^ii^	1.509 (10)
C4—H4A	0.9300		
			
O2—Zn—O4	99.5 (2)	H7A—C7—H7B	120.0
O2—Zn—N4	110.1 (2)	C7—C8—C9	129.8 (12)
O4—Zn—N4	107.7 (2)	C7—C8—H8A	115.1
O2—Zn—N2	106.0 (2)	C9—C8—H8A	115.1
O4—Zn—N2	110.6 (2)	N3—C9—C8	113.7 (8)
N4—Zn—N2	120.8 (3)	N3—C9—H9A	108.8
C19—O2—Zn	116.2 (5)	C8—C9—H9A	108.8
C20—O4—Zn	110.6 (5)	N3—C9—H9B	108.8
C6—N1—C4	107.3 (6)	C8—C9—H9B	108.8
C6—N1—C3	125.9 (8)	H9A—C9—H9B	107.7
C4—N1—C3	126.7 (7)	C11—C10—N3	106.2 (7)
C6—N2—C5	105.3 (7)	C11—C10—H10A	126.9
C6—N2—Zn	126.9 (6)	N3—C10—H10A	126.9
C5—N2—Zn	127.3 (5)	C10—C11—N4	110.6 (8)
C12—N3—C10	108.0 (7)	C10—C11—H11A	124.7
C12—N3—C9	125.7 (7)	N4—C11—H11A	124.7
C10—N3—C9	126.1 (7)	N4—C12—N3	109.6 (7)
C12—N4—C11	105.6 (7)	N4—C12—H12A	125.2
C12—N4—Zn	125.7 (5)	N3—C12—H12A	125.2
C11—N4—Zn	127.7 (5)	C14—C13—C18	122.3 (7)
C2—C1—H1A	120.0	C14—C13—H13A	118.8
C2—C1—H1B	120.0	C18—C13—H13A	118.8
H1A—C1—H1B	120.0	C13—C14—C15	119.0 (8)
C1—C2—C3	125.2 (15)	C13—C14—H14A	120.5
C1—C2—H2A	117.4	C15—C14—H14A	120.5
C3—C2—H2A	117.4	C16—C15—C14	120.7 (8)
N1—C3—C2	109.5 (8)	C16—C15—H15A	119.6
N1—C3—H3A	109.8	C14—C15—H15A	119.6
C2—C3—H3A	109.8	C15—C16—C17	121.5 (7)
N1—C3—H3B	109.8	C15—C16—H16A	119.2
C2—C3—H3B	109.8	C17—C16—H16A	119.2
H3A—C3—H3B	108.2	C16—C17—C18	118.0 (7)
C5—C4—N1	107.0 (7)	C16—C17—C20^i^	117.6 (6)
C5—C4—H4A	126.5	C18—C17—C20^i^	124.4 (6)
N1—C4—H4A	126.5	C13—C18—C17	118.2 (7)
C4—C5—N2	109.2 (8)	C13—C18—C19	121.6 (7)
C4—C5—H5A	125.4	C17—C18—C19	120.1 (6)
N2—C5—H5A	125.4	O1—C19—O2	123.3 (7)
N1—C6—N2	111.1 (8)	O1—C19—C18	121.3 (6)
N1—C6—H6A	124.4	O2—C19—C18	115.4 (7)
N2—C6—H6A	124.4	O3—C20—O4	124.7 (7)
C8—C7—H7A	120.0	O3—C20—C17^ii^	118.6 (7)
C8—C7—H7B	120.0	O4—C20—C17^ii^	116.2 (7)
			
O4—Zn—O2—C19	−175.7 (5)	C10—N3—C9—C8	92.3 (11)
N4—Zn—O2—C19	71.4 (5)	C7—C8—C9—N3	2(2)
N2—Zn—O2—C19	−60.9 (5)	C12—N3—C10—C11	−0.7 (10)
O2—Zn—O4—C20	171.3 (5)	C9—N3—C10—C11	−175.6 (8)
N4—Zn—O4—C20	−73.9 (5)	N3—C10—C11—N4	−0.7 (10)
N2—Zn—O4—C20	60.1 (5)	C12—N4—C11—C10	1.9 (10)
O2—Zn—N2—C6	30.4 (7)	Zn—N4—C11—C10	170.5 (6)
O4—Zn—N2—C6	137.3 (6)	C11—N4—C12—N3	−2.2 (9)
N4—Zn—N2—C6	−95.6 (7)	Zn—N4—C12—N3	−171.2 (5)
O2—Zn—N2—C5	−140.5 (7)	C10—N3—C12—N4	1.9 (9)
O4—Zn—N2—C5	−33.6 (8)	C9—N3—C12—N4	176.8 (8)
N4—Zn—N2—C5	93.5 (8)	C18—C13—C14—C15	2.4 (12)
O2—Zn—N4—C12	123.9 (7)	C13—C14—C15—C16	−0.3 (13)
O4—Zn—N4—C12	16.3 (7)	C14—C15—C16—C17	−3.0 (13)
N2—Zn—N4—C12	−112.1 (7)	C15—C16—C17—C18	4.2 (11)
O2—Zn—N4—C11	−42.7 (7)	C15—C16—C17—C20^i^	−178.6 (8)
O4—Zn—N4—C11	−150.2 (7)	C14—C13—C18—C17	−1.0 (11)
N2—Zn—N4—C11	81.4 (7)	C14—C13—C18—C19	176.9 (7)
C6—N1—C3—C2	127.2 (11)	C16—C17—C18—C13	−2.2 (10)
C4—N1—C3—C2	−54.2 (13)	C20^i^—C17—C18—C13	−179.2 (7)
C1—C2—C3—N1	−119.3 (14)	C16—C17—C18—C19	179.9 (7)
C6—N1—C4—C5	0.7 (11)	C20^i^—C17—C18—C19	2.8 (10)
C3—N1—C4—C5	−178.1 (9)	Zn—O2—C19—O1	−8.2 (9)
N1—C4—C5—N2	0.4 (12)	Zn—O2—C19—C18	171.1 (4)
C6—N2—C5—C4	−1.4 (11)	C13—C18—C19—O1	−159.2 (7)
Zn—N2—C5—C4	171.1 (7)	C17—C18—C19—O1	18.8 (10)
C4—N1—C6—N2	−1.6 (10)	C13—C18—C19—O2	21.6 (10)
C3—N1—C6—N2	177.2 (8)	C17—C18—C19—O2	−160.5 (6)
C5—N2—C6—N1	1.8 (10)	Zn—O4—C20—O3	7.5 (9)
Zn—N2—C6—N1	−170.7 (5)	Zn—O4—C20—C17^ii^	−164.5 (4)
C12—N3—C9—C8	−81.8 (11)		

Symmetry codes: (i) −*x*+3/2, *y*, *z*−1/2; (ii) −*x*+3/2, *y*, *z*+1/2.

### Hydrogen-bond geometry (Å, °)

**Table:**

*D*—H···*A*	*D*—H	H···*A*	*D*···*A*	*D*—H···*A*
C3—H3A···O2^i^	0.97	2.56	3.424 (10)	148
C5—H5A···O3	0.93	2.43	3.070 (10)	126
C6—H6A···O1	0.93	2.38	3.080 (11)	132
C9—H9A···O3^iii^	0.97	2.48	3.317 (11)	144

Symmetry codes: (i) −*x*+3/2, *y*, *z*−1/2; (iii) *x*−1/2, −*y*+1, *z*.
